# Biomineral Precursor Formation Is Initiated by Transporting Calcium and Phosphorus Clusters from the Endoplasmic Reticulum to Mitochondria

**DOI:** 10.1002/advs.201902536

**Published:** 2020-03-02

**Authors:** Cuizhu Tang, Yan Wei, Lin Gu, Qinghua Zhang, Mei Li, Guohua Yuan, Yuan He, Li Huang, Yan Liu, Yufeng Zhang

**Affiliations:** ^1^ State Key Laboratory Breeding Base of Basic Science of Stomatology (Hubei‐MOST) and Key Laboratory of Oral Biomedicine Ministry of Education School and Hospital of Stomatology Wuhan University 237 Luoyu Road Wuhan 430079 China; ^2^ Medical Research Institute School of Medicine Wuhan University Wuhan 430071 China; ^3^ Beijing National Laboratory for Condensed Matter Physics Institute of Physics Chinese Academy of Sciences Beijing 100190 China; ^4^ Key Laboratory of Catalysis and Materials Science of the State Ethnic Affairs Commission and Ministry of Education College of Chemistry and Materials Sciences College of Resources and Environmental Science South‐Central University for Nationalities Wuhan 430074 China; ^5^ Medical Research Center for Structural Biology School of Basic Medical Sciences Wuhan University Wuhan 430071 China; ^6^ Laboratory of Biomimetic Nanomaterials Department of Orthodontics Peking University School and Hospital of Stomatology 22# Zhongguancun South Avenue, Haidian District Beijing 100081 China

**Keywords:** biomineralization, black phosphorus, endoplasmic reticulum, mineral precursors, mitochondrion

## Abstract

Mineral granules in the mitochondria of bone‐forming cells are thought to be the origin of biomineral precursors, which are transported to extracellular matrices to initiate cell‐mediated biomineralization. However, no evidence has revealed how mitochondrial granules form. This study indicates that mitochondrial granules are initiated by transporting calcium and phosphorus clusters from the endoplasmic reticulum (ER) to mitochondria based on detailed observations of the continuous process of mouse parietal bone development as well as in vitro biomineralization in bone‐forming cells. Nanosized biomineral precursors (≈30 nm in diameter), which originate from mitochondrial granules, initiate intrafibrillar mineralization of collagen as early as embryonic day 14.5. Both in vivo and in vitro studies further reveal that formation of mitochondrial granules is induced by the ER. Elevated levels of intracellular calcium or phosphate ions, which can be induced by treatment with ionomycin and black phosphorus, respectively, accelerate formation of the calcium and phosphorus clusters on ER membranes and ultimately promote biomineralization. These findings provide a novel insight into biomineralization and bone formation.

## Graphical abstract

The origin of mineral granules in the mitochondria, which are considered as biomineral precursors involved in the biomineralization process, has not been revealed. This study indicates that mitochondrial granules are initiated by transporting calcium and phosphorus clusters from the endoplasmic reticulum to mitochondria. Elucidation of the natural development of biomineral precursors is essential for a better understanding of biomineralization.

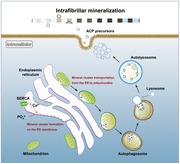

## 1 Introduction

Biomineralization is a ubiquitous and tightly regulated process that is fundamental to human bone development and health. Accumulating evidence shows that the formation of amorphous calcium phosphate (ACP) precursors is crucial for biomineralization in vertebrates.^1^ Over the past 50 years, a series of studies conducted in various organisms, such as the mammalian skeleton,^2^ zebrafish fin rays,^3^ and coccolithophorid alga,[qv: 1c] have unveiled the existence of ACP precursors. However, the origin of the ACP precursors remains unclear.

In previous in vivo studies, researchers focus mostly on already mineralized bone and deduce matrix vesicles (MVs) (30–1000 nm in diameter) as an initiator of biomineralization.^4^ Nevertheless, many natural bone formation details remain uncertain, including i) the factor(s) that originally initiates the collagen mineralization process, MVs or not, and ii) the origin of biomineral precursors, including MVs. Recent in vitro cell culture studies demonstrate that the electron‐dense granules residing in the mitochondria of mineralizing cells are intracellular ACP precursors.^5^ Boonrungsiman et al.[qv: 5a] observe direct transportation of calcium ions and possibly phosphate ions between mitochondria and intracellular vesicles. Another two studies[qv: 5b,6] demonstrate that ACP precursors are transferred from mitochondria via the lysosomal pathway. Until now, observations on the continuous in vivo process of biomineralization are lacking, and there is still no evidence revealing the mechanisms of formation of the mitochondrial granules.

Unrevealing the process of biomineral precursor formation is essential for understanding biomineralization and bone formation. Here, we investigated the process of biomineralization during natural bone development to elucidate the origin of biomineral precursors. Moreover, to explore the underlying mechanisms of intracellular precursor formation, ionomycin and black phosphorus nanosheets (BPs) were used to increase the concentrations of intracellular calcium and phosphate ions in cultured bone‐forming cells.

## 2 Results and Discussion

### 2.1 The Process of Collagen Mineralization in Developing Mouse Parietal Bone and Dentine

**Figure 1:**
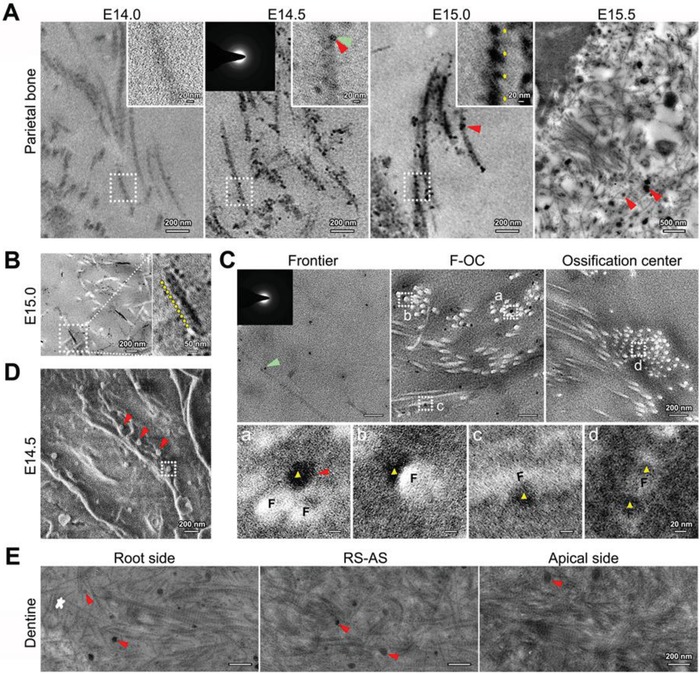
Nanoscale observations of collagen mineralization in developing mouse parietal bone and dentine. A,B) Unstained TEM images of collagen mineralization in parietal bone. Images in (B) were taken in the area closer to the ossification center compared with that in (A, E15.0). Red triangles: electron‐dense biomineral precursors; right inset: higher magnification of delineated collagen microfibrils; left inset: selected area electron diffraction (SAED) of a mineral precursor at E14.5, indicated by green triangles; yellow arrows: periodic banding of microfibrils. C) Unstained TEM images of collagen and biomineral precursors at E14.5. F‐OC: area between the frontier and ossification center of the bone. Inset: SAED of a mineral precursor indicated by the green triangle; F: collagen microfibrils; red triangles: biomineral precursors; yellow triangles: precursors attached to a,b) the microfibril surface or infiltrating into c,d) the microfibrils. D) SEM of parietal bone at E14.5. Red triangles: biomineral precursors. E) Unstained TEM images of collagen mineralization in dentine at E18.0. White star: the frontier of dentine; RS‐AS: area between the root side and apical side; red triangles: biomineral precursors.

We first evaluated extracellular collagen mineralization in developing mouse calvarial bone. Embryos of C57BL/6J mice were collected, and the expression of alkaline phosphatase (ALP), an early marker of osteoblastic differentiation,^7^ was examined. Ossification centers of the parietal bone were visible by ALP staining on embryonic day (E)14.0 (Figure S1, Supporting Information). Transmission electron microscopy (TEM) observations showed that tropocollagen molecules were secreted into the extracellular space, and microfibrils (37.59 ± 0.95 nm in width) without mineralization formed at E14.0 (**Figure** 1A). Besides, periodic banding of the microfibrils at this stage was a little vague. At E14.5, amorphous electron‐dense particles (26.68 ± 1.61 nm in diameter) formed, arranged in or closely around microfibrils, and initiated intrafibrillar mineralization of collagen. Periodic banding of 67.22 ± 1.33 nm became obvious and corresponded to the *D*‐periods of collagen molecules by E15.0 (Figure 1A,B). At E15.5, mineralized collagen fibrils formed and were also surrounded by large‐sized electron‐dense particles (100–200 nm in diameter). By E18.0, highly mineralized and parallel fibril arrays formed, with mineral apatites oriented parallel to the longitudinal axis of the fibrils (Figure S2A, Supporting Information). Moreover, interfibrillar mineralization was observed at this stage (Figure S2B, Supporting Information). Taken together, the collagen mineralization process was initiated from the amorphous mineral phase to the ordered alignment of mineral apatites.

The freeze‐substitution method for TEM sample preparation was applied to clarify the association between the mineral particles and collagen microfibrils. The results showed that electron‐dense amorphous particles were spread over the extracellular matrix at the bone frontier, and more particles were assembled around microfibrils in the area close to the ossification center at E14.5 (Figure 1C). Furthermore, some particles were attached to the surface of collagen microfibrils, and some seemed to have infiltrated into them. These observations provided a strong evidence for the liquid state of biomineral precursors.^1^, ^2^, ^3^, ^8^ Additionally, scanning electron microscopy (SEM) images revealed that most of the particles were distributed closely around the microfibrils (Figure 1D). These particles were confirmed to be MVs, as energy dispersive X‐ray spectroscopy (EDS) analysis revealed an aggregation of calcium, with a higher calcium‐to‐phosphorus ratios found for the particles closer to the ossification center (Figure S3, Supporting Information). Taken together, hierarchical associations between collagen and biomineral precursors were discerned at the nanoscale level during intrafibrillar collagen mineralization.^9^ This study might provide a basis for theoretical studies of collagen mineralization, such as osmotic equilibrium,^10^ ACP precursors,^11^ and water‐oriented apatite crystals.^12^ Similar results were obtained during tooth development, as small‐sized mineral particles were first observed among collagen fibrils, and larger particles were observed later (Figure 1E). However, whether the size of the particles has an influence on collagen mineralization was not clear. Mahamid et al.^3^, ^13^ reported that mineral particles adhering to collagen microfibrils have diameters of 10–20 nm. Our previous in vitro study also reported that MVs (100–200 nm in diameter) can be divided into smaller ones (±30 nm) and the latter promoted osteoblastic differentiation more remarkably.^14^ All of these results imply that intrafibrillar mineralization may be mediated directly by biomineral precursors with diameters of 10–30 nm.

### 2.2 Evolution of Granule‐Deposited Mitochondria during Mouse Parietal Bone Development

**Figure 2:**
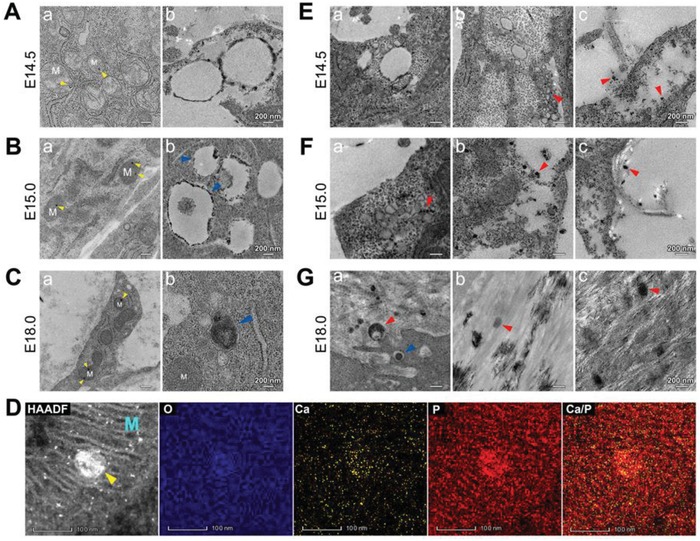
TEM observations of mitochondrial evolution during mouse parietal bone development. A–C) Unstained TEM images of the mitochondria in osteoblasts. a) Mitochondria deposited with electron‐dense granules (yellow triangles). b) Vacuolar mitochondria in (A,B) and agglomerated mitochondria in (C). M: the mitochondria; blue triangles: single‐membrane autolysosomes in (B) and a double‐membrane autophagosome in (C). D) High‐angle annular dark‐field scanning transmission electron microscopy (HAADF‐STEM) EDS elemental mapping of a mitochondrial granule (yellow triangles). M: the mitochondria. E–G) TEM images of biomineral precursors (red triangles) being transported out of cells at E) E14.5 (stained), F) E15.0 (stained), and G) E18.0 (unstained). Blue triangle: a single‐membrane autolysosome.

The origin of extracellular biomineral precursors was further explored. It remains uncertain if the process is mediated by resident cells and, if so, whether the biomineral precursors form intra‐ or extracellularly. Apart from bone‐forming cells, blood vessels are the most relevant source of biomineral precursors, as angiogenesis and osteogenesis occur almost simultaneously in bone,^15^ and biomineral particles exist in blood vessels.^16^ By E14.5, blood vessels in bone had not yet fully formed (Figure S4, Supporting Information). Nevertheless, the morphological structure of mitochondria in osteoblasts clearly changed during the process of bone development (**Figure** 2; Figure S5, Supporting Information). The initial changes in mitochondria were deposits of electron‐dense granules of ≈30 nm in diameter (Figure 2A–C). The EDS elemental mapping images showed a dense cluster of calcium and phosphorus in the core of the granules (Figure 2D). At E14.5 and E15.0, mitochondria appeared to vacuolate and electron‐dense granules aggregated on or close to the mitochondrial membranes. These vacuolated mitochondria were identified in single‐membrane autolysosomes, which broke into debris along with biomineral precursors of ≈10–40 nm in diameter (Figure 2E,F). More electron‐dense granules were observed in the mitochondria at E18.0. Mitochondrial vacuolization was rarely observed at this stage. Instead, the mitochondria became electron‐dense masses and eventually developed into large‐sized biomineral precursors, undergoing a transportation process of mitophagy (Figure 2G). It appeared that smaller‐sized precursors were produced first, which was consistent with a previous study.^6^ Despite the time sequence, all biomineral precursors originated from mitochondria. The mechanisms of formation of the mitochondrial granules were further explored.

### 2.3 ER–Mitochondria Associations in Osteoblasts during Mouse Parietal Bone Development

**Figure 3:**
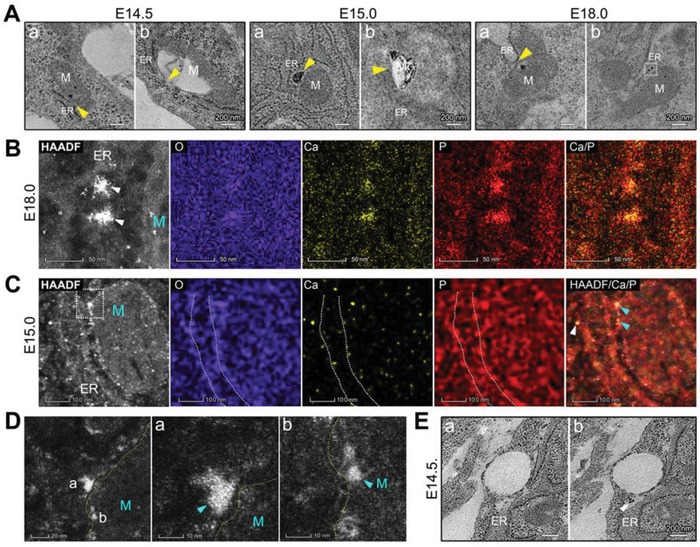
TEM observations of ER–mitochondria associations in osteoblasts during mouse parietal bone development. A) TEM images of ER–mitochondria (M) associations (yellow triangles) at E14.5 (stained), E15.0 (unstained), and E18.0 (unstained). B) HAADF‐STEM EDS elemental mapping of the electron‐dense granules on ER membranes (white triangles) in (A, the outlined area). M: the mitochondria. C) HAADF‐STEM EDS elemental mapping of an osteoblast at E15.0. M: the mitochondria; white triangles: electron‐dense granules on or around the ER membranes (white‐dotted line); aquamarine triangles: electron‐dense granules on the outer mitochondrial membranes. D) HAADF‐STEM images of the outlined area in (C) Yellow‐dotted line: the outer mitochondrial membrane. E) Stained TEM images of two serial sections of parietal bone at E14.5. White triangle: granules on or around the ER.

According to the observations of initially altered mitochondria, the mitochondria always changed from a local area, and interestingly, these areas were always close to the rough endoplasmic reticulum (ER) (**Figure** 3). We inferred that formation of mitochondrial granules was induced by the ER possibly via the following ways: i) proteins located on the ER regulated the process in mitochondria;^17^ ii) inorganic ions were transported from the ER;^18^ iii) calcium and phosphorus clusters were transported from the ER. The mitochondria in developing osteoblasts were closely surrounded by ER, and a higher electron density of granules on ER membranes was observed compared with undifferentiated cells (Figure S6, Supporting Information). At E18.0, even particular calcium and phosphorus clusters, as dense as the mitochondrial granules, were observed on ER membranes (Figure 3A,B). Some electron‐dense granules of ≈10 nm in diameter located around the ER, and some granules located on the outer mitochondrial membranes close to the ER at E15.0 (Figure 3C,D). At E14.5, electron‐dense granules on the ER membranes were not obvious, but some electron‐dense granules were observed between the ER and vacuolar mitochondria (Figure 3E). All the above findings indicated that formation of the mitochondrial granules was initiated by transporting clusters of calcium and phosphorus from the ER. The functions of the ER in biomineralization were confirmed in vitro.

### 2.4 In Vitro Biomineralization Process in Cultured Bone‐Forming Cells

**Figure 4:**
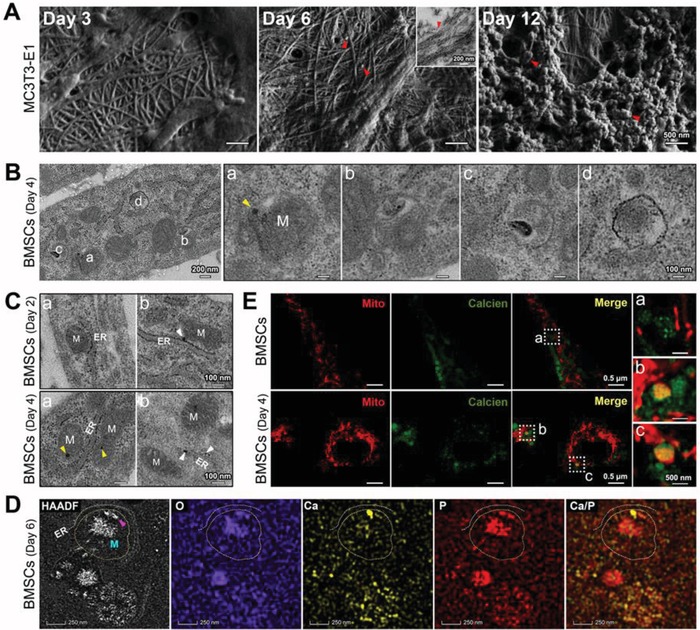
Nanoscale observations of the mineralization process in cultured bone‐forming cells. A) SEM of collagen fibrils and biomineral precursors (red triangles) secreted by MC3T3‐E1 cells after 3, 6, and 12 days of osteoinduction. Inset: an unstained TEM image. B) Unstained TEM images of the mitochondria (M) in BMSCs after 4 days of osteoinduction. Yellow triangles: mitochondrial granules. C) Unstained TEM images of ER–mitochondria associations in BMSCs after 2 or 4 days of osteoinduction. M: the mitochondria; yellow triangles: mitochondrial granules; white triangles: electron‐dense granules on or around the ER. D) HAADF‐STEM EDS elemental mapping of mitochondria (M) in BMSCs after 6 days of osteoinduction. Purple triangle: calcium aggregation within a mitochondrion close to the ER; yellow‐dotted line: the outer mitochondrial membrane; white‐dotted line: the ER membrane. E) Confocal microscopy images of untreated BMSCs and BMSCs after 4 days of osteoinduction. The mitochondria and calcium were stained with mitochondrial‐Tracker (red) and calcein‐AM (green).

Bone‐forming cells, the mouse osteoblast precursor cell line MC3T3‐E1, and bone marrow‐derived mesenchymal stem cells (BMSCs), were evaluated during biomineralization processes, including collagen mineralization (**Figure** 4A; Figure S7, Supporting Information), mitochondrial evolution (Figure 4B; Figure S8, Supporting Information), and ER–mitochondria associations (Figure 4C,D; Figure S9, Supporting Information). Figure 4B shows a consecutive transformation process of the mitochondria in BMSC‐induced osteoblasts. Moreover, mitophagy was observed (Figures S8 and S10, Supporting Information), similar to the in vitro study by Pei et al.[qv: 5b] Additionally, local permeability changes in the outer mitochondrial membranes were confirmed by confocal microscopy. Calcein‐AM, a conventional fluorescence indicator of intracellular calcium ions, cannot normally pass through the mitochondrial membrane. However, some mitochondria in osteoblasts were partially stained by calcein‐AM (Figure 4E). We deduced that some macromolecules, such as the calcium and phosphorus clusters, might be transferred into mitochondria.

### 2.5 Effects of Intracellular Calcium Ion Elevation on BMSC‐Induced Osteoblasts

**Figure 5:**
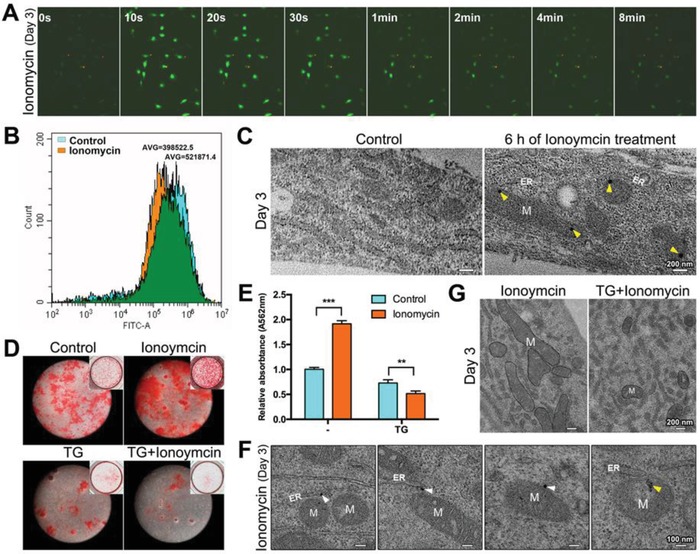
Enhanced formation of mitochondrial granules by intracellular [Ca^2+^]_i_ elevation. A) Confocal microscopy images of intracellular calcium in osteoblasts treated with ionomycin for 8 min. Calcium was indicated by Fluo‐4. B) Flow cytometry analysis of intracellular [Ca^2+^]_i_ in osteoblasts with or without (Control) 6 h of ionomycin treatment. AVG: average fluorescence value. C) Unstained TEM images of mitochondria (M) in osteoblasts with or without 6 h of ionomycin treatment. Yellow triangles: mitochondrial granules. D) ARS staining of BMSCs after 9 days of osteoinduction. E) Relative quantification of ARS staining in (D). **p < 0.01; ***p < 0.001. F) Unstained TEM images of the transportation process of electron‐dense granules from the ER to mitochondria (white triangles) in osteoblasts after 6 h of ionomycin treatment. Yellow triangles: mitochondrial granules. G) Unstained images of the mitochondria (delineated, M) after 6 h of ionomycin treatment in osteoblasts with or without TG stimulation. The osteoblasts originated from BMSCs after 3 days of osteoinduction.

Ionomycin was used to increase the concentrations of intracellular calcium ions ([Ca^2+^]_i_). The dynamic changes in [Ca^2+^]_i_ of BMSC‐induced osteoblasts were monitored using the Fluo‐4 calcium ion indicator. Consistent with a previous study,^19^ there was a rapid increase in the [Ca^2+^]_i_ within the first 30 s of ionomycin stimulation (**Figure** 5A). However, after 6 h of treatment, the [Ca^2+^]_i_ decreased compared with the untreated cells, as revealed by flow cytometry (Figure 5B). More electron‐dense granules were observed in the mitochondria (Figure 5C). The decrease in [Ca^2+^]_i_ might be attributed to formation of these granules. Ionomycin effectively facilitated the mineralization of osteoblasts as revealed by Alizarin Red‐S (ARS) staining (Figure 5D,E). Moreover, after 6 h of ionomycin treatment, we observed granules transported from the ER membranes to the mitochondria (Figure 5F). It has been shown that the mitochondria and the ER are closely associated in osteoblasts, and that ionomycin‐induced calcium ions are concentrated close to these regions.^17^ Whether the formation of ionomycin‐induced mitochondrial granules is related to calcium uptake by the ER needs further investigation. Sarco[endo]plasmic reticulum calcium ATPase (SERCA) plays a key role in calcium uptake by the ER, and meanwhile, thapsigargin (TG) is a specific inhibitor of SERCA.^20^ A low dose of TG, which does not elicit detectable changes in the cytosolic [Ca^2+^]_i_,^21^ was used prior to ionomycin treatment in osteoblasts. The shape of the mitochondria changed from long rod to short rod or spheres in TG‐treated cells, but no/few electron‐dense granules were observed on ER membranes or in mitochondria (Figure 5G). ARS staining indicated that ionomycin no longer promoted, but rather inhibited, mineralization of osteoblasts. Taken together, biomineralization enhanced by the cytosolic [Ca^2+^]_i_ might be mediated directly by the ER, not mitochondria, and calcium uptake by the ER might be essential for the formation of mitochondrial granules.

### 2.6 Effects of Intracellular Phosphorus Ion Elevation on BMSCs and BMSC‐Induced Osteoblasts

**Figure 6:**
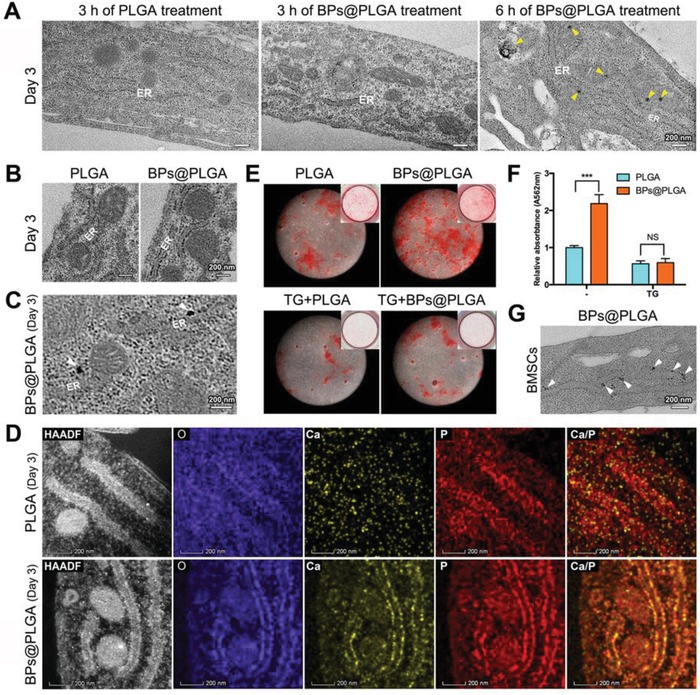
Calcium and phosphorus cluster transportation from the ER to the mitochondria. A) Unstained TEM images of PLGA‐ or BPs@PLGA‐treated osteoblasts. Yellow triangles: mitochondrial granules. B,C) Unstained TEM images of osteoblasts after 3 h of PLGA or BPs@PLGA treatment. White triangles: electron‐dense granules on the ER. D) HAADF‐STEM EDS elemental mapping of osteoblasts after 3 h of PLGA or BPs@PLGA treatment. E) ARS staining of BMSCs after 9 days of osteoinduction. F) Relative quantification of ARS staining in (E). ***p < 0.001; NS: not significant. G) Unstained TEM images of BMSCs after 3 h of BPs@PLGA treatment. White triangles: electron‐dense granules on the ER. The osteoblasts originated from BMSCs after 3 days of osteoinduction.

Next, the form of inorganic ion transportation from the ER to mitochondria was further explored. According to previous studies, black phosphorus shows great potential for bone repair or regeneration, which is attributed to its photothermal effect and supply of phosphate ions.^22^ However, how phosphate ions work, by participating in biomineralization intra‐ versus extracellularly, is unknown. Since bare black phosphorus exhibits intrinsic instability and degrades rapidly in the extracellular environment, appropriate black phosphorus passivation strategies should be considered to increase intracellular phosphorus.^23^ In this study, poly(lactic‐*co*‐glycolic acid) (PLGA) was applied to enclose the BPs (named BPs@PLGA, Scheme S1, Supporting Information), which was conducive to endocytosis. These nanospheres rapidly entered BMSC‐induced osteoblasts within 30 min, as revealed by confocal microscopy (Figure S11, Supporting Information). A number of mitochondrial granules were observed after 6 h of BPs@PLGA treatment (**Figure** 6A). Nevertheless, within 3 h of BPs@PLGA treatment, many electron‐dense granules were located on the ER membranes (Figure 6B), and some were as large as ≈20 nm in diameter (Figure 6C). The EDS mapping images revealed numerous clusters of calcium and phosphorus on the ER (Figure 6D). Furthermore, BPs@PLGA effectively promoted mineralization of BMSC‐induced osteoblasts (Figure 6E,F). However, in the case of pretreatment with TG, BPs@PLGA no longer promoted cell mineralization, and only a few mitochondrial granules were observed (Figure S12, Supporting Information). This finding further confirmed that calcium uptake by the ER is a prerequisite for the formation of mitochondrial granules. Similarly, BPs@PLGA also promoted osteoblastic differentiation and formation of electron‐dense granules on ER membranes in BMSCs (Figure 6G; Figure S13, Supporting Information). Many mitochondrial granules were observed in BMSCs after 6 h of BPs@PLGA treatment, and the EDS elemental mapping showed a very dense cluster of phosphorus (Figure S14, Supporting Information). Thus, not only calcium but also phosphorus could be transported from the ER to the mitochondria.

## 3 Conclusion

**Scheme 1:**
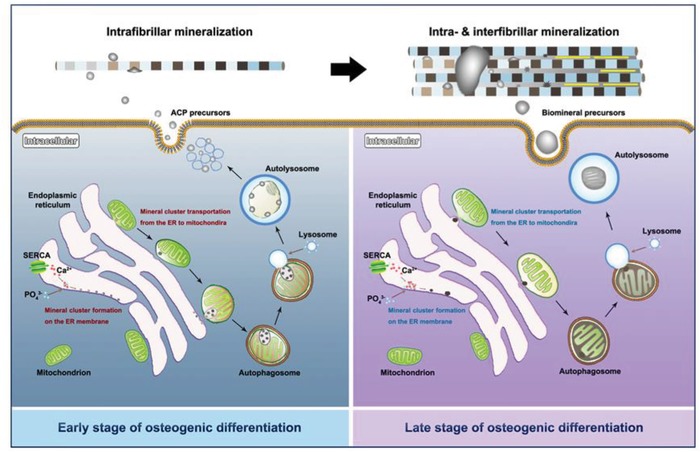
Schematic illustration of biomineralization process and mechanisms underlying biomineral precursor formation.

The present study proposes that the ER is the initiation site of mitochondrial mineral precursors according to detailed observations of mouse parietal bone development (**Scheme** 1). Furthermore, calcium uptake by the ER is essential for biomineralization. Calcium and phosphorus clusters are generated on the ER membranes and are transported into the mitochondria. Biomineral precursors eventually form during mitochondrial evolution. Additionally, ≈30 nm diameter biomineral precursors appear to directly initiate intrafibrillar mineralization. This study reveals the natural development of biomineral precursors and is essential for a better understanding of bone formation and biomineralization.

## Conflict of Interest

The authors declare no conflict of interest.

## Supporting information

Supporting Information

## References

[1] a) J. J. De Yoreo , P. U. Gilbert , N. A. Sommerdijk , R. L. Penn , S. Whitelam , D. Joester , H. Zhang , J. D. Rimer , A. Navrotsky , J. F. Banfield , A. F. Wallace , F. M. Michel , F. C. Meldrum , H. Colfen , P. M. Dove , Science 2015, 349, aaa6760; 26228157 10.1126/science.aaa6760

[2] N. J. Crane , V. Popescu , M. D. Morris , P. Steenhuis , M. A. Ignelzi Jr. , Bone 2006, 39, 434. 16627026 10.1016/j.bone.2006.02.059

[3] J. Mahamid , A. Sharir , L. Addadi , S. Weiner , Proc. Natl. Acad. Sci. USA 2008, 105, 12748. 18753619 10.1073/pnas.0803354105 PMC2529085

[4] a) M. Murshed , Cold Spring Harbor Perspect. Med. 2018, 8, a031229; 10.1101/cshperspect.a031229 PMC6280711 29610149

[5] a) S. Boonrungsiman , E. Gentleman , R. Carzaniga , N. D. Evans , D. W. Mccomb , A. E. Porter , M. M. Stevens , Proc. Natl. Acad. Sci. USA 2012, 109, 14170; 22879397 10.1073/pnas.1208916109 PMC3435222

[6] T. Iwayama , T. Okada , T. Ueda , K. Tomita , S. Matsumoto , M. Takedachi , S. Wakisaka , T. Noda , T. Ogura , T. Okano , P. Fratzl , T. Ogura , S. Murakami , Sci. Adv. 2019, 5, eaax0672. 31281900 10.1126/sciadv.aax0672 PMC6609213

[7] U. Sharma , D. Pal , R. Prasad , Indian J. Clin. Biochem. 2014, 29, 269. 24966474 10.1007/s12291-013-0408-y PMC4062654

[8] a) M. J. Olszta , X. Cheng , S. S. Jee , R. Kumar , Y.‐Y. Kim , M. J. Kaufman , E. P. Douglas , L. B. Gower , Mater. Sci. Eng., R 2007, 58, 77;

[9] Y. Liu , D. Luo , X. Kou , X. Wang , F. R. Tay , Y. Sha , Y. Gan , Y. Zhou , Adv. Funct. Mater. 2013, 23, 1404.

[10] L. Niu , S. E. Jee , K. Jiao , L. Tonggu , M. Li , L. Wang , Y. Yang , J. Bian , L. Breschi , S. S. Jang , J. Chen , D. H. Pashley , F. R. Tay , Nat. Mater. 2017, 16, 370. 27820813 10.1038/nmat4789 PMC5321866

[11] a) Y. Wang , T. Azais , M. Robin , A. Vallee , C. Catania , P. Legriel , G. Pehau‐Arnaudet , F. Babonneau , M. M. Giraud‐Guille , N. Nassif , Nat. Mater. 2012, 11, 724; 22751179 10.1038/nmat3362

[12] Y. Wang , S. V. Euw , F. M. Fernandes , S. Cassaignon , M. Selmane , G. Laurent , G. Pehau‐Arnaudet , C. Coelho , L. Bonhomme‐Coury , M. M. Giraud‐Guille , F. Babonneau , T. Azaïs , N. Nassif , Nat. Mater. 2013, 12, 1144. 24193662 10.1038/nmat3787

[13] J. Mahamid , B. Aichmayer , E. Shimoni , R. Ziblat , C. Li , S. Siegel , O. Paris , P. Fratzl , S. Weiner , L. Addadi , Proc. Natl. Acad. Sci. USA 2010, 107, 6316. 20308589 10.1073/pnas.0914218107 PMC2851957

[14] Y. Wei , C. Tang , J. Zhang , Z. Li , X. Zhang , R. J. Miron , Y. Zhang , Biochem. Biophys. Res. Commun. 2019, 514, 252. 31029430 10.1016/j.bbrc.2019.04.029

[15] a) S. K. Ramasamy , A. P. Kusumbe , L. Wang , R. H. Adams , Nature 2014, 507, 376; 24647000 10.1038/nature13146 PMC4943529

[16] M. Kerschnitzki , A. Akiva , A. B. Shoham , N. Koifman , E. Shimoni , K. Rechav , A. A. Arraf , T. M. Schultheiss , Y. Talmon , E. Zelzer , S. Weiner , L. Addadi , Bone 2016, 83, 65. 26481471 10.1016/j.bone.2015.10.009

[17] A. A. Rowland , G. K. Voeltz , Nat. Rev. Mol. Cell Biol. 2012, 13, 607. 22992592 10.1038/nrm3440 PMC5111635

[18] C. Giorgi , S. Marchi , P. Pinton , Nat. Rev. Mol. Cell Biol. 2018, 19, 713. 30143745 10.1038/s41580-018-0052-8

[19] C. Bordat , J. L. Guerquin‐Kern , M. Lieberherr , G. Cournot , Histochem. Cell Biol. 2004, 121, 31. 14673658 10.1007/s00418-003-0601-9

[20] Y. G. Zhao , Y. Chen , G. Miao , H. Zhao , W. Qu , D. Li , Z. Wang , N. Liu , L. Li , S. Chen , P. Liu , D. Feng , H. Zhang , Mol. Cell 2017, 67, 974. 28890335 10.1016/j.molcel.2017.08.005

[21] N. Engedal , M. L. Torgersen , I. J. Guldvik , S. J. Barfeld , D. Bakula , F. Sætre , L. K. Hagen , J. B. Patterson , T. Proikas‐Cezanne , P. O. Seglen , A. Simonsen , I. G. Mills , Autophagy 2014, 9, 1475. 10.4161/auto.25900 23970164

[22] a) X. Wang , J. Shao , M. A. Raouf , H. Xie , H. Huang , H. Wang , P. K. Chu , X. Yu , Y. Yang , A. M. Abdel‐Aal , N. H. Mekkawy , R. J. Miron , Y. Zhang , Biomaterials 2018, 179, 164; 29986234 10.1016/j.biomaterials.2018.06.039

[23] a) H. Wang , K. Hu , Z. Li , C. Wang , Z. Li , Small 2018, 14, 1801701; 10.1002/smll.201801701 30084541

