# Supplementary material for: Biomineral Precursor Formation Is Initiated by Transporting Calcium and Phosphorus Clusters from the Endoplasmic Reticulum to Mitochondria
Source: Adv Sci (Weinh). 2020 Mar 2;7(8):1902536. doi: 10.1002/advs.201902536 (PMC7175271; doi:10.1002/advs.201902536)
Supplement: Supplementary file 1 — Supporting Information [file ADVS-7-1902536-s001.pdf]

Copyright WILEY-VCH Verlag GmbH & Co. KGaA, 69469 Weinheim, Germany, 2019.

Supporting Information

for *Adv. Sci.,* DOI: 10.1002/advs.201902536

Biomineral Precursor Formation Is Initiated by Transporting Calcium and Phosphorus Clusters from the Endoplasmic Reticulum to Mitochondria

Cuizhu Tang, Yan Wei, Lin Gu, Qinghua Zhang, Mei Li, Guohua Yuan, Yuan He, Li Huang, Yan Liu, and Yufeng Zhang**

Copyright WILEY-VCH Verlag GmbH & Co. KGaA, 69469 Weinheim, Germany, 2019.

Supporting Information

Biomineral Precursor Formation is Initiated by Transporting Calcium and Phosphorus Clusters from the Endoplasmic Reticulum to Mitochondria

Cuizhu Tang, Yan Wei, Lin Gu, Qinghua Zhang, Mei Li, Guohua Yuan, Yuan He, Li Huang, Yan Liu, Yufeng Zhang**

Experimental Section

Animals

C57BL/6J mice were purchased from Center for Disease Control of Hubei province. All surgical procedures used in these experiments were approved by the Ethics Committee for Animal Research, Wuhan University, China (ethics approval number: 2017/69).

Histological alkaline phosphatase (ALP) staining

Pregnant mice were anesthetized and the embryos were extracted out. The embryonic calvarial bones were fixed by 4% paraformaldehyde (pH 7.2-7.4) for 36 h, and then made into paraffin sections by using standard methods. The expression of ALP in embryonic parietal bones was examined with NBT/BCIP Alkaline Phosphatase Color Development Kit (Beyotime, China).

Transmission electron microscopy (TEM) analysis

The TEM samples were prepared with the support of Research Center for medicine and structural biology, Wuhan University, China. Briefly, embryonic calvarial bones and cultured cells were fixed with 2.5% glutaraldehyde for 2 h at room temperature. Then, the cells were scraped from culture dishes and the cell suspensions were centrifuged at 1000 rpm for 5 min. The obtained precipitate was enclosed by agarose to keep the scattered cells as a whole. Subsequently, embryonic calvarial bones and cell clusters were fixed in 1% Osmic acid (AR) (Sinopharm Chemical Reagent Co., Ltd., China), dehydrated by ethanol and acetone series, and embedded in epoxy resin.

The freeze-substitution method was also used for sample preparation as described previously.[1] Briefly, embryonic calvarial bones were immersed in 50% dimethyl sulfoxide at 4 °C for 24 h, freeze-substituted in 4% Osmic acid dissolved in acetone at -80 °C for 2 days, at -20 °C for 2 h, at 4 °C for 2 h, and at room temperature for 2 h. Then, the tissues were embedded in epoxy resin.

70-nm ultrathin sections, with or without uranyl acetate and lead citrate staining, were imaged by using a HT7700 transmission electron microscope (Hitachi, Japan) at 80 kV. The electron diffraction patterns of selected areas were recorded by using a Technai 20 transmission electron microscope (Philips, Japan) at 120 kV. High-angle annular dark-field (HAADF) analysis and energy dispersive X-ray spectroscopy (EDS) elemental mapping were performed using a Cs-corrected JEM ARM200F scanning transmission electron microscope (JEOL, Japan) at 200 kV.

Scanning electron microscopy (SEM) analysis

Embryonic samples were made into 10-μM paraffin sections. after xylene dewaxing for 2 h, the

sections were treated with gold sputtering and then examined using a MERLIN Compact field-emission scanning electron microscope (Zeiss, Germany) at 120 kV. Besides, cultured MC3T3-E1 cells were fixed with 2.5% glutaraldehyde for 2 h at room temperature and then dehydrated by ethanol series. After the gold sputtering treated, the samples were imaged and characterized by EDS coupled to SEM.

Preparation of nanospheres incorporating black phosphorus nanosheets (BPs)

Poly(lactic-co-glycolic acid) (PLGA) is an ideal drug delivery system and has been approved by the Food and Drug Administration. To increase intracellular phosphorus, PLGA (50:50, MW: 30000-60000) (Sigma-Aldrich, USA) was applied to enclose BPs by using a liquid exfoliation method as described previously.[2] For synthesis of BPs, the black phosphorus crystal powder (XFNANO, China) was added to N-methyl-2-pyrrolidone (Aladdin, China) at a concentration of 1 mg mL^{-1}. The mixture was then sonicated in an ice bath for 14 h, including 8 h of 2-s period with 0.1-s internal and 6 h of 2-s period with 4-s internal. Subsequently, the solution was centrifuged at 4000 rpm for 10 min to remove the un-exfoliated particles. 10 mL of the obtained supernatant was centrifuged for another 20 min at 12000 rpm, and the precipitate was redispersed in 1 mL of dichloromethane (DCM) (Sigma-Aldrich, USA), which contained PLGA with a concentration of 10 mg mL^{-1}. Then, the BPs and PLGA mixture was sonicated for 15 min and added into 10 mL of 0.5% (w/v) polyvinyl alcohol (Sigma-Aldrich, USA). The obtained emulsion was stirred overnight at room temperature, followed by being centrifuged at 7000 rpm for 10 min and being washed with PB buffer solution (PBS) for three times. Finally, the PLGA nanospheres incorporating BPs (named BPs@PLGA) were prepared successfully.

The nanospheres were further conjugated with a FITC-labeled ssDNA (FITC-ssDNA).[3] Briefly, carbodiimide hydrochlorid (EDC) and hydroxysuccinimide (NHS) (Sigma-Aldrich, USA) were mixed with a ratio of 1:1.5, and 1 mL of the nanospheres in PBS were added into the EDC/NHS solution and agitated at room temperature for 30 min to activate the carboxyl groups on the surface of these nanospheres. After three times of PBS washing, the nanospheres in PBS were reacted with the FITC-ssDNA at room temperature for 3 h. Lastly, the fluorescence-labeled PLGA and BPs@PLGA nanospheres were purified by washing with PBS twice.

In vitro osteogenic differentiation

Bone marrow-derived mesenchymal stem cells (BMSCs) were isolated from the tibias and femurs of 4-6-week-old C57BL/6J mice as previously reported.[4] All experiments were performed at cell passages 3-6. For osteogenic induction, osteogenic differentiation medium containing 10 nM of dexamethasone, 10 mM of β-glycerol phosphate and 0.2 mM of ascorbic acid (Sigma-Aldrich, USA) substituted the growth medium at 80% cell confluence. To increase the concentrations of intracellular calcium or phosphorus ions, cells were treated with ionomycin (5 μM) (Beyotime, China) or BPs@PLGA (50 ppm) for 6 h. In the case of blocking calcium uptake by the ER, 100 nM of thapsigargin (TG)[5] (Sigma-Aldrich, USA) was used to treat BMSC-induced osteoblasts for 2 h prior to ionomycin or BPs@PLGA treatment.

Alizarin Red-S (ARS) staining was performed to detect matrix mineralization deposition. MC3T3-E1 cells (ATCC, USA) and BMSCs were osteoinduced for approximately 21 and 9 days, respectively. Afterwards, the cells were fixed with 95% alcohol and then stained with 0.2% ARS

(pH 4.2). For quantitative analysis, the stain was dissolved by 10% cetylpyridinium chloride and assayed at 562 nm by spectrophotoetry.

Live-cell imaging

Local permeability changes in the outer mitochondrial membranes of osteoblasts were studied by confocal microscopy. The osteoblasts originated from BMSCs after 4 days of osteoinduction, and BMSCs without osteoinduction served as a control. These two groups of cells were cultured in medium containing calcein-AM (1 μg mL^{-1}) (Dojindo, Japan) for 12 h. Subsequently, the cells were stained with mitochondrial-Tracker (1:10000) (Thermo Fisher Scientific, USA) for 15 min at 37 °C according to the manufacturer's instruction. Live-cell imagining was performed with a LCS SP8 STED confocal microscope (Leica microsysterms, Germany).

Intracellular calcium ions were indicated by Fluo-4 AM (Beyotime, China). Osteoblasts originating from BMSCs after 3 days of oseoinduction were incubated with fluo-4 AM (1 μM) for 30 min at 37 °C. Real-time monitoring of intracellular calcium levels in osteoblasts under the condition of ionomycin (5 μM) treatment was performed with the Leica confocal microscope.

The uptake of BPs@PLGA by osteoblasts was also observed by confocal microscopy. The osteoblasts, originating from BMSCs after 3 days of osteoinductin, were imaged, following 30 min of fluorescence-labeled BPs@PLGA (10 ppm) treatment.

Flow cytometry assays

Intracellular calcium levels were quantified using Fluo-4 AM in untreated osteoblasts and in osteoblasts treated with ionomycin. The osteoblasts originated from BMSCs after 3 days of

osteoinduction. After 6 h of ionomycin (5 μM) treatment, cells were trypsinized. Then, $1x10^5$ cells of each group were collected and incubated with Fluo-4 AM (1 μM) for 30 min at 37 °C. Average fluorescence values of the two groups were detected by a Aria III flow cytometer (BD, America).

To determine whether the nanospheres binded with the FITC-ssDNA, flow cytometry assay was used to measure the fluorescence values of unmodified PLGA and fluorescence-labeled PLGA.

Statistical analysis

All data were collected from repeated experiments and presented as mean ± standard deviation. Student's *t* test and one-way analysis of variance were performed for the comparison among groups. Results were considered significant at $p < 0.05$.

Supporting Scheme and Figures

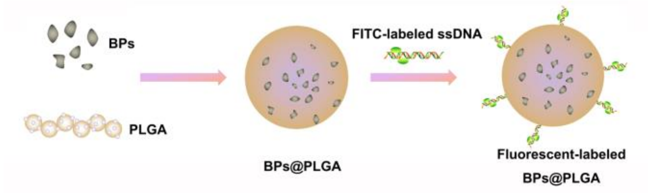

Scheme S1. Schematic illustration of BPs@PLGA nanosphere formation.

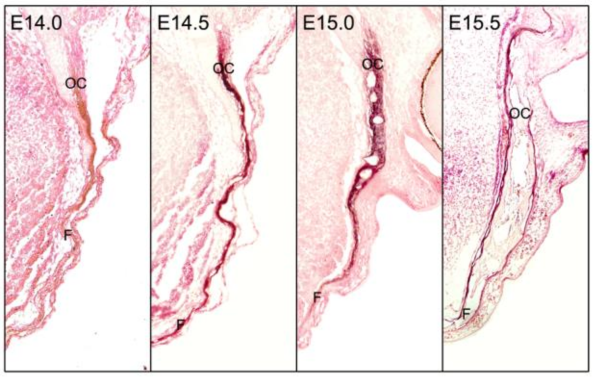

Figure S1. ALP staining of developing mouse parietal bone on embryonic day (E)14.0, E14.5, E15.0, and E15.5. OC: the ossification center; F: the frontier. It has been reported that the aggregation and osteogenic differentiation of bone-forming cells in mouse craniofacial bone started roughly at E13.5.[6] Therefore, ossification centers of the parietal bone were already observed at E14.0.

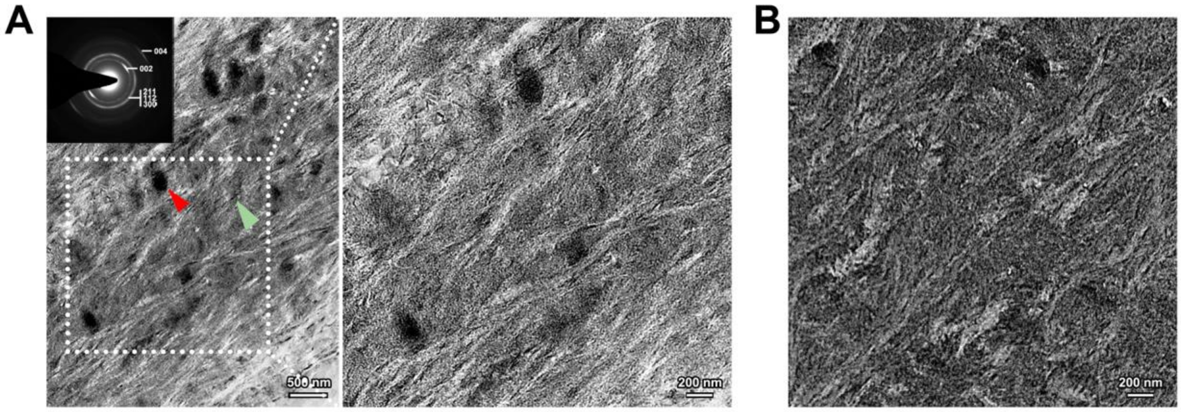

Figure S2. Unstained TEM images of collagen mineralization in parietal bone at E18.0. **A**) Intrafibrillar mineralization close to the frontier of bone. Red triangles: biomineral precursors; left inset: selected area electron diffraction (SAED), indicated by a green triangle. **B**) Intra- and interfibrillar mineralization at the ossification center of bone.

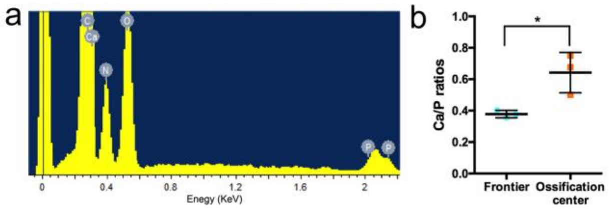

Figure S3. a) SEM-EDS analysis of the nano-sized mineral particles delineated in Figure 1D. b) SEM-EDS analysis of the calcium-to-phosphorus (Ca/P) ratio of the mineral particles at the frontier and the ossification center of mouse parietal bone at E14.5. *: $p < 0.05$.

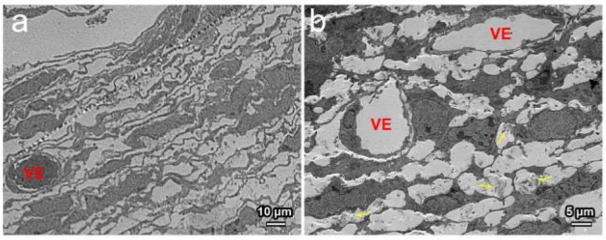

Figure S4. Stained TEM images of blood vessels at the frontier (a) and ossification center (b) of mouse parietal bone at E14.5. VE: vessels. Yellow arrows: extracellular collagen fibrils.

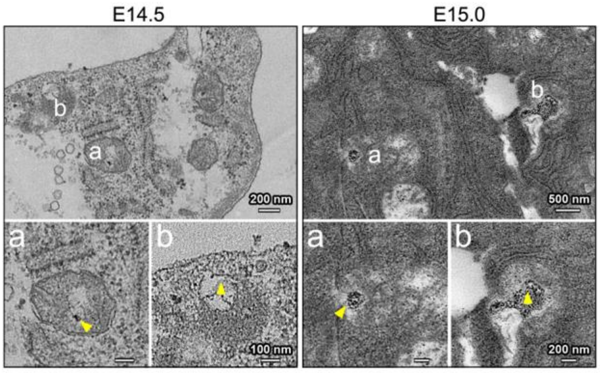

Figure S5. Unstained TEM images of mitochondria (before vacuolization) in osteoblasts at E14.5 and E15.0. Yellow triangles: electron-dense deposits in mitochondria.

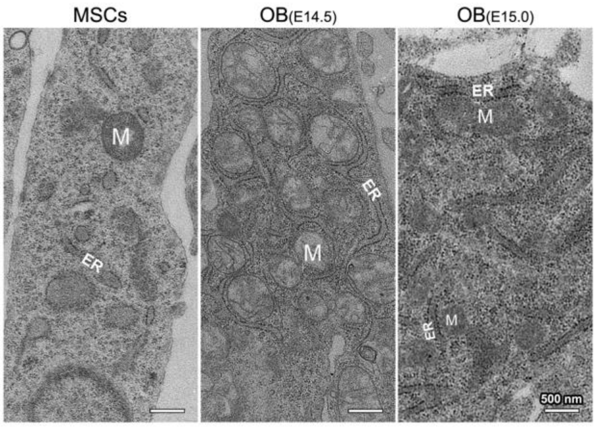

Figure S6. TEM images of undifferentiated BMSCs (stained) and osteoblasts (OBs) (unstained) at E14.5 and E15.0.

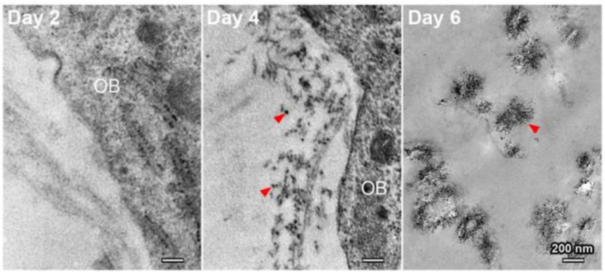

Figure S7. Unstained TEM images of extracellular biomineral precursors (red triangles) from osteoblasts (OBs). The cells originated from BMSCs after 2, 4, or 6 days of osteoinduction.

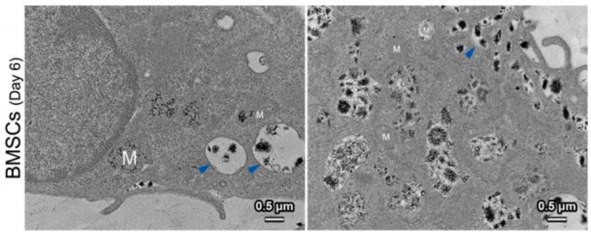

Figure S8. Unstained TEM images of mitochondira in BMSCs after 6 days of osteoinduction. M: the mitochondria; blue triangles: single-membrane autolysosomes.

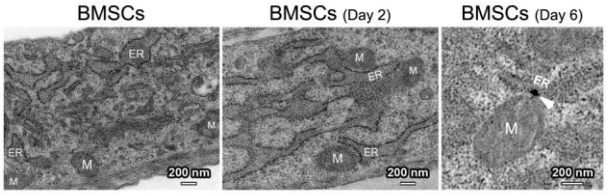

Figure S9. TEM images of untreated BMSCs (stained) and BMSCs after 2 or 6 days of osteoinduction (unstained). M: the mitochondria; white triangle: an electron-dense granule located on the ER membrane.

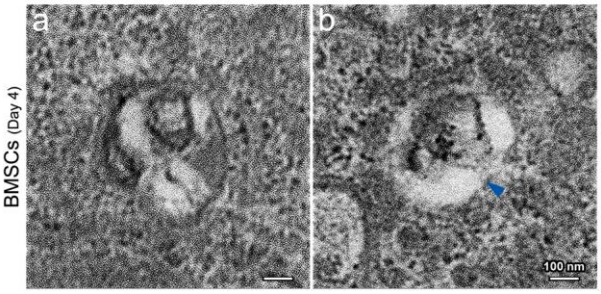

Figure S10. Unstained TEM images of mitophagy in BMSCs after 4 days of osteoinduction. a) A mitochondrion with internal electron-dense deposits. b) A single-membrane autolysosome (blue triangle) containing a degraded mitochondrion with electron-dense deposits.

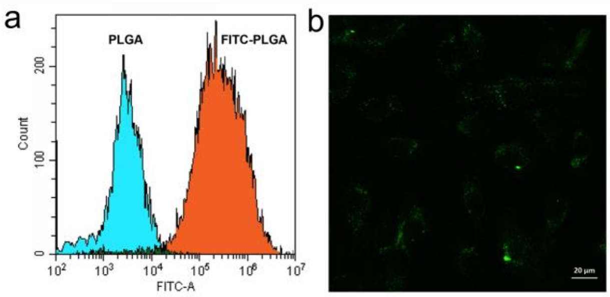

Figure S11. a) Quantified fluorescence values of unmodified PLGA and fluorescence-labeled PLGA (FITC-PLGA) by flow cytometry. b) A confocal microscopy image of BMSC-induced osteoblasts after FITC-PLGA incubation for 30 m.

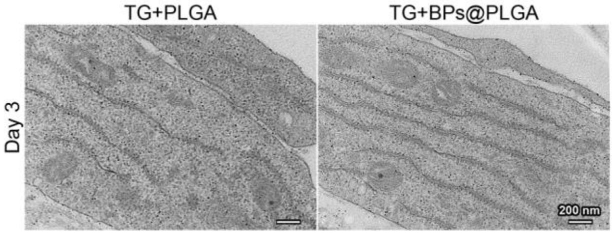

Figure S12. Unstained TEM images of osteoblasts treated with PLGA or BPs@PLGA for 6 h following 2 h of TG stimulation. The osteoblasts originated from BMSCs after 3 days of osteoinduction.

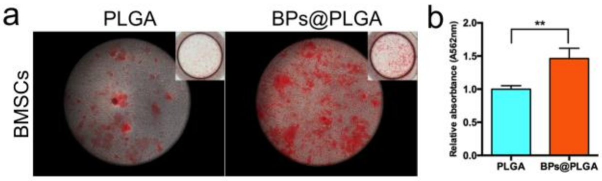

Figure S13. Elevation of intracellular phosphorus levels induced by BPs@PLGA promoted the mineralization of BMSCs. a) ARS staining of BMSCs after 9 days of osteoinduction. BMSCs were treated with BPs@PLGA for 6 h before osteoinduction. b) Relative quantification of ARS staining in (a). **: $p < 0.01$.

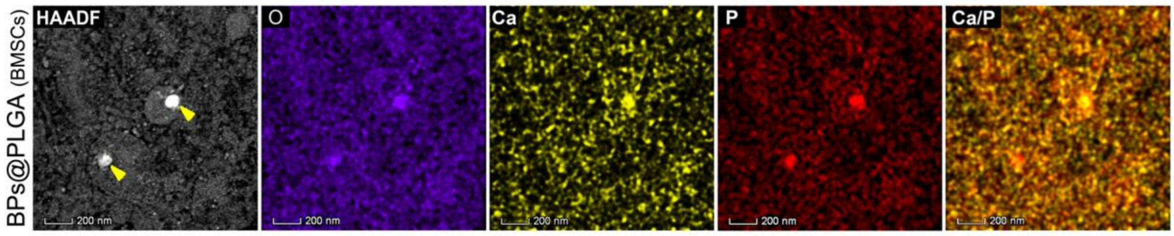

Figure S14. HAADF-STEM EDS elemental mapping of mitochondrial granules (yellow triangle) in BMSCs after BPs@PLGA treatment for 6 h.

References

[1]K. Hoshi, S. Kemmotsu, Y. Takeuchi, N. Amizuka, H. Ozawa, *J B Miner Res* **2010**, *14,* 273.

[2]a）Z. Sun, H. Xie, S. Tang, X. Yu, Z. Guo, J. Shao, H. Zhang, H. Huang, H. Wang, P. K. Chu, *Angew. Chem., Int. Ed. Engl.* **2015**, *54*, 11526; b）X. Wang, J. Shao, M. a. E. Raouf, H. Xie, Y. Zhang, *Biomaterials* **2018**, *179*, 164; c）Y. Wang, X. Hu, L. Zhang, C. Zhu, J. Wang, Y. Li, Y. Wang, C. Wang, Y. Zhang, Q. Yuan, *Nat. Commun.* **2019**, *10*, 2829; d）J. Shao, H. Xie, H. Huang, Z. Li, Z. Sun, Y. Xu, Q. Xiao, X. Yu, Y. Zhao, H. Zhang, *Nat. Commun.* **2016**, *7*, 12967.

[3]X. Hu, Y. Wang, Y. Tan, J. Wang, H. Liu, Y. Wang, S. Yang, M. Shi, S. Zhao, Y. Zhang, *Adv. Mater.* **2017**, *29*, 1605235.

[4]S. Liu, D. Liu, C. Chen, K. Hamamura, A. Moshaverinia, R. Yang, Y. Liu, Y. Jin, S. Shi, *Cell Metab.* **2015**, *22*, 606.

[5]a）N. Engedal, M. L. Torgersen, I. J. Guldvik, S. J. Barfeld, D. Bakula, F. Sætre, L. K. Hagen, J. B. Patterson, T. Proikas-Cezanne, P. O. Seglen, A. Simonsen, I. G. Mills, *Autophagy* **2014**, *9*, 1475; b）Y. G. Zhao, Y. Chen, G. Miao, H. Zhao, W. Qu, D. Li, Z. Wang, N. Liu, L. Li, S. Chen, P. Liu, D. Feng, H. Zhang, *Mol. Cell* **2017**, *67*, 974.

[6]a）T. Sasaki, Y. Ito, P. B. Jr, S. Chou, M. M. Urata, H. Slavkin, Y. Chai, *Development* **2006**, *133*, 371; b）M. Hosokawa, M. Shoji, K. Kitamura, T. Tanaka, T. Noce, S. Chuma, N. Nakatsuji, *Dev. Bio.* **2006**, *301*, 0.
